# Hypercapnia: is it protective in lung injury?

**DOI:** 10.1186/2045-9912-3-23

**Published:** 2013-11-11

**Authors:** Alexander F Bautista, Ozan Akca

**Affiliations:** 1 Department of Anesthesiology & Perioperative Medicine, University of Louisville, Louisville, KY 40202, USA; 2 Outcomes ResearchTM Consortium, Cleveland Clinic Foundation, Cleveland, OH, USA

**Keywords:** Hypercapnia, *Hypercarbia*, Carbon Dioxide, Oxygenation, Acute Lung Injury (ALI), Ventilator Associated Lung Injury (VALI), Pneumonia, Sepsis, Acute Respiratory Distress Syndrome (ARDS), Ventilator-induced diaphragmatic dysfunction (VIDD)

## Abstract

Hypercapnic acidosis has been regarded as a tolerated side effect of protective lung ventilation strategies. Various in vivo and ex vivo animal studies have shown beneficial effects in acute lung injury setting, but some recent work raised concerns about its anti-inflammatory properties. This mini-review article aims to expand the potential clinical spectrum of hypercapnic acidosis in critically ill patients with lung injury. Despite the proven benefits of hypercapnic acidosis, further safety studies including *dose-effect*, *level-and-onset of anti-inflammatory effect*, and *safe applicability period* need to be performed in various models of lung injury in animals and humans to further elucidate its protective role.

## Introduction

Management of critically ill patients has been continuously evolving. Technological advancement has improved the immediacy and quality of monitoring. Basic and translational researches have changed the approach in medical management from simplistic acid–base approach to much more sophisticated real-time compartmental electrolyte assessments to neurally-adjusted ventilator management. Now, we can follow inhaled-exhaled oxygen and carbon dioxide levels at our bedside patient monitors, and assess blood gas partial pressures as our stat labs.

Hypercapnia with and without acidosis has been observed in critically ill patients for many decades. This observation was either regarded as *not beneficial* or even *harmful* mostly due to its pH-related effects and complicated responses in the central nervous system. Recent studies have shown compelling evidence suggesting beneficial effects of hypercapnic acidosis in various different lung injury models in animals and human [1-3]. The majority of basic and clinical work focused on ventilatory strategies with regards to the beneficial effects of moderate hypercapnia. This article aims to discuss the beneficial and *potentially* harmful effects of permissive hypercapnia in the perspective of critically ill patients with lung injury.

### Effects on heart, lungs, & oxygenation

#### Carbon dioxide & cellular compartments

Carbon dioxide (CO_2_) is a by-product of cellular respiration. It plays a major role in the acid–base balance essential for intracellular and extracellular hemostasis. It has moderate water solubility. The reaction of dissolved CO_2_ and water will yield carbonic acid, with the enzyme *carbonic anhydrase* reversibly catalyzing the dissociation of carbonic acid:

(1) $$H_{2}0+CO_{2}\to H_{2}CO_{3}\to H^{+}+\mathrm{HC}{O_{3}}^{-}\to H^{+}+C{O_{3}}^{2-}$$

Carbon dioxide moves across the intracellular compartment via a concentration gradient. It is actively removed through exhalation by the respiratory system. Changes in partial pressure of CO_2_ will effect changes in hydrogen ion concentration.

#### Clinically established levels of carbon dioxide

The normal carbon dioxide tension is considered to be within the range of 35–45 mmHg. Hypercapnia is generally defined as carbon dioxide partial pressure of higher than 45 mmHg. *Mild hypercapnia* is noted to having CO_2_ partial pressure of up to 50 mmHg [4,5]. Jung et. al, defined *moderate hypercapnia* as PaCO_2_ levels between the range of 55 to 70 mmHg [3]. Levels higher than 75 mmHg are generally regarded as *severe hypercapnia*[6,7]. When considering these arbitrary levels of hypercapnia, it is important to emphasize that severity leveling of hypercapnia alone without a corresponding pH value, which is also affected by metabolic changes, may not represent the expected frame of clinical effects.

Since there is an overlap between the partial CO_2_ ranges presented in most of the clinical studies cited in this article, we are alluding to *mild-to-moderate hypercapnia* for discussion purposes. Significance and contribution of acidosis levels are not equal between the studies cited. Therefore, we tried to provide the contribution of acidosis for each listed study.

#### Respiratory effects of hypercapnia

The main physiologic effect of increased CO_2_ in patients would result in a right-ward shift of the oxygen dissociation curve which, in effect, improves unloading of oxygen at the tissue level. In a canine model, arterial hypercapnia produced a gradual and significant increase in oxygen carrying capacity [8]. An increase in intracellular H^+^ led to decrease in pulmonary vascular resistance [9] abolishing hypoxic pulmonary vasoconstriction, reduction in intrapulmonary shunting and improvement of ventilation-perfusion mismatch [10]. Hypercapnia also attenuates fluid dynamics in pulmonary vasculature, which would keep the diffusion distance between the pulmonary capillary and the alveoli short, thereby preserving optimal gas exchange and preventing edema formation [11]. Although hypercapnia may notably increase pulmonary vascular resistance, due to its overall effects in decreasing pro-inflammatory response and fluid dynamics in the pulmonary vasculature, the overall effect may not always appear as increased pulmonary vascular resistance.

#### Effects on hemodynamics and oxygenation

Hypercapnia increases cardiac output, oxygen carrying capacity, mixed venous oxygen, and also peripheral tissue oxygenation [12-15]. The increases in cerebral tissue oxygenation can be observed to some extent even when cardiac output was kept constant [16] which can possibly be explained by the relationship of oxygen supply and demand. Increase in cardiac output appears to be related to inotropic effect through ß-adrenergic receptors, and hypercapnia-induced sympathetic activation and catecholamines [17,18]. Hypercapnia decreases systemic vascular resistance, [19] which may further support tissue perfusion especially when intravascular volume status is maintained.

Cardiac index increases by about 10-15% for each 10-mmHg increments in arterial carbon dioxide tension within a range of 30 to 50 mmHg [12,20]. Additionally, the rightward shift of the oxyhemoglobin dissociation curve and decreases in systemic vascular resistance increase oxygen availability to the peripheral tissue [20]. Ratnaraj *et al.* showed that increasing end-tidal carbon dioxide tension from 30 to 50 mmHg improved both subcutaneous tissue oxygen tension by ~ 23%, and intramural oxygenation in large and small intestine by 16-to-45% under general anesthesia in a pig model [21]. Fleischmann *et al*. showed that even under high inspired oxygen concentration of 0.80 in colorectal surgery patients who were assigned to mild hypercapnia (ET PCO_2_ of 50 mmHg), subcutaneous tissue oxygenation increased by 38% and colon intramural oxygenation increased by about 100% compared to the patients who were assigned to normocapnia (ET PCO_2_ of 35 mmHg) [4]. More recently, Schwartges *et al.* elegantly showed that incremental levels of carbon dioxide increased cardiac output and systemic oxygen delivery in dogs, which was measured as dissolved oxygen and gastric mucosal oxygen saturation [22].

In spite of the positive oxygenation and perfusion effects by hypercapnia as described above, it should also be noted that myocardial depression due to overwhelming effects on inotropy and catecholamines may develop at CO_2_ concentrations greater than 10-15% (i.e., PaCO_2_ > 75 mmHg) [23,24] regardless of compensatory mechanisms to correct acidosis.

#### Hypercapnia & ventilator-associated lung injury (VALI)

Mechanical stress associated with overstretching alveoli can cause lung injury. This is clearly evident with the use of higher tidal volumes and increased transpulmonary pressure during mechanical ventilation resulting in **v**entilator **a**ssociated **l**ung **i**njury (VALI). Experimental studies have shown that lung injury markers such as the presence of pulmonary edema, hyaline membranes, epithelial injury, filtration coefficient [25] and lymphatic flow are increased with higher tidal volumes [26]. To minimize this insult, it has been advocated to utilize smaller tidal volumes i.e. 7 ml/kg or less [27] and to limit the plateau airway pressure to 35 cm H_2_O or lower [28]. This in effect will cause a slow increase in PaCO_2_ hence implementation of permissive hypercapnia if there is no adjustment in respiratory rate [2]. In a prospective randomized animal study with moderate to severe ventilation-induced lung injury, hypercapnic acidosis significantly reduced stretch-induced lung injury and resulted in higher arterial PO_2_ compared to normocapnia. Moreover, it also showed reduction in lung permeability as evidenced by bronchoalveolar protein concentrations and attenuated the decrease in static lung compliance in comparison with the normocapnia group [29]. This study confirms the growing body of evidence that suggests hypercapnic acidosis involvement in the inhibition of nuclear factor kappa-B (NF-KB) that regulates genes central to lung injury, inflammation and repair.

In a large, randomized-controlled trial from the ARDS Network, Kregenow and colleagues demonstrated that permissive hypercapnia (pH < 7.35 and PCO2 > 45 mmHg) reduced 28-day mortality in patients with acute lung injury who were ventilated with 12 mL/kg predicted body weight tidal volume [30]. This is in part due the ability of hypercapnic acidosis to attenuate physiologic and histological indices of lung injury induced by very high levels of lung stretch, [31] but not that due to the collapse and re-expansion of atelectatic lung [1]. Laffey and Kavanagh also stated that the combination of reduction of lung stretch and permissive hypercapnia is additive in preventing VALI [32].

#### Hypercapnia & ventilator-induced diaphragmatic injury (VIDD)

Ventilator-induced diaphragmatic dysfunction (VIDD) is another complication of prolonged controlled ventilation. It may progress to diaphragmatic muscle atrophy and result in long-term dependence on mechanical ventilation [33]. Diaphragmatic muscle weakening can be explained by proteolysis, apoptosis and oxidative stress, [34] and it appears to be directly proportional to duration of mechanical ventilation; [35] anti-oxidant and anti-inflammatory agents such as steroids may attenuate the process [36,37].

Recently, Jung et al. reported that after 72 hours of controlled mechanical ventilation, diaphragm contractions to maximal stimulus were preserved in hypercapnic animals compared to a 25% decrease in the normocapnic ventilated animals [38]. Controlling lung injury, preventing diaphragmatic dysfunction, and altering exaggerated pro-inflammatory responses may suggest an important potential role for mild-to-moderate hypercapnia and hypercapnic acidosis in acutely developing systemic inflammatory processes [39].

#### Hypercapnia in acute lung injury (ALI) and acute respiratory distress syndrome (ARDS)

The causes of ALI and ARDS are varied, however, the mainstay in the management of both disease processes is mechanical ventilation, which can by itself further result in VALI as described above. Hence, protective ventilator strategy with permissive hypercapnia has been studied both in human and animal models. Ischemia-reperfusion injury is highly associated with the development of ALI. Shibata et al. reported that hypercapnic acidosis inhibits xanthine oxidase, and therefore prevents an increase in capillary permeability, attenuates free-radical-induced lung injury, and possibly prevents apoptosis [40]. Other mechanisms by which hypercapnic acidosis provide protection include attenuation of key etiologic factors that lead to ALI, reduction of physical lung damage, inhibition of key aspects of the inflammatory response and direct protection of systemic organs [41]. As mentioned above, the data published by the ARDS Network and others have shown that hypercapnic acidosis in this subset of patients had a reduced mortality [27,28,30].

#### Hypercapnia, pneumonia & sepsis

Pneumonia and sepsis are the leading causes of increased morbidity in critically ill patients [42]. Hypercapnic acidosis has shown to depress immune function through various mechanisms via macrophage suppression, inhibition of phagocytic function of neutrophils and cytokine signaling [43,44]. However, most of these data reported in animal studies and *in vitro* models. Curley et al. stated that hypercapnic acidosis inhibits the activation of NF-KB, [29]which is responsible for activation and regulation of pro-inflammatory and repair processes suggesting the reduction of pulmonary epithelial wound healing [45].

Such poor healing phenomenon was demonstrated in *in vitro* studies using human bronchial epithelial cells exposed to hypercapnia at neutral pH environment [46]. Despite this mechanistic effect of hypercapnia on the immune system, hypercapnic acidosis has been shown to improve physiologic indices of injury in the setting of acute bacterial pneumonia [47]. Ni Chonghaile et. al. demonstrated that hypercapnic acidosis attenuated the increase in airway pressure and the decrease in both lung compliance and arterial oxygenation in their animal model via a neutrophil-independent mechanism [47]. In a follow-up study, using their established pneumonia model, they showed that the protective effects of hypercapnic acidosis are further enhanced with the use of appropriate antibiotic therapy [48]. On the contrary, in prolonged untreated pneumonia animal model, hypercapnic acidosis appeared to worsen lung indices in terms of static compliance and arterial oxygenation, [49] and was associated with higher bacterial count and increased mortality [50].

With regard to systemic sepsis, animal models with hypercapnic acidosis demonstrated improvement in hemodynamic parameters, preserved central venous oxygen saturation, slowed the development of hypotension, and attenuated the associated increase in lactic acid concentration when compared to normocapnia [51-53]. Additionally, hypercapnia may have some minimal effect in preventing other infection such as surgical site infections [54]. The clinical implication of hypercapnia in effect is also dependent on the stage of the infection, i.e. early or late in the course of the disease process; and also the primary site of infection, may it be pulmonary or systemic source.

## Summary

In conclusion, moderate hypercapnia may play a protective role and may be an integral part of ventilatory management of critically ill patients with VALI, VIDD, ALI and ARDS. Regarding its role in immunomodulation, it may be protective in the setting of acute pneumonia and sepsis, provided adequate antibiotic coverage has been instituted in a timely fashion. However, it should be noted that moderate hypercapnia may worsen the injury in prolonged, undiagnosed, untreated pneumonia. Further clinical trials in animals, healthy human subjects, and patients are needed to further assess the safety of hypercapnia, to find the optimum dosing and timing, and finally to elucidate its overall protective role.

## Competing interests

The authors and institution have no conflict of interests. They have no financial or personal relationships with other people or organization that could inappropriately influence their action.

## Authors’ contributions

OA and AFB conceptualized the idea and wrote the manuscript. Both authors have read and approved the final manuscript.
